# Cytoplasmic CXCR4 expression in breast cancer: induction by nitric oxide and correlation with lymph node metastasis and poor prognosis

**DOI:** 10.1186/1471-2407-8-340

**Published:** 2008-11-23

**Authors:** Hironao Yasuoka, Masahiko Tsujimoto, Katsuhide Yoshidome, Masaaki Nakahara, Rieko Kodama, Tokio Sanke, Yasushi Nakamura

**Affiliations:** 1 Department of Clinical Laboratory Medicine, Wakayama Medical University, Wakayama, Japan; 2 Department of Pathology, Osaka Police Hospital, Osaka, Japan; 3 Department of Surgery, Osaka Police Hospital, Osaka, Japan

## Abstract

**Background:**

Lymph nodes constitute the first site of metastasis for most malignancies, and the extent of lymph node involvement is a major criterion for evaluating patient prognosis. The CXC chemokine receptor 4 (CXCR4) has been shown to play an important role in lymph node metastasis. Nitric oxide (NO) may also contribute to induction of metastatic ability in human cancers.

**Methods:**

CXCR4 expression was analyzed in primary human breast carcinoma with long-term follow-up. The relationship between nitrotyrosine levels (a biomarker for peroxynitrate formation from NO in vivo) and lymph node status, CXCR4 immunoreactivity, and other established clinico-pathological parameters, as well as prognosis, was analyzed. Nitrite/nitrate levels and CXCR4 expressions were assessed in MDA-MB-231 and SK-BR-3 breast cancer cell lines after induction and/or inhibition of NO synthesis.

**Results:**

CXCR4 staining was predominantly cytoplasmic; this was observed in 50%(56/113) of the tumors. Cytoplasmic CXCR4 expression was significantly correlated with nitrotyrosine levels and lymph node metastasis. Kaplan-Meier survival curves showed that cytoplasmic CXCR4 expression was associated with reduced disease-free and overall survival. In multivariate analysis, cytoplasmic CXCR4 expression emerged as a significant independent predictor for overall and disease-free survival. Cytoplasmic expression of functional CXCR4 in MDA-MB-231 and SK-BR-3 cells was increased by treatment with the NO donor DETA NONOate. This increase was abolished by L-NAME, an inhibitor of NOS.

**Conclusion:**

Our data showed a role for NO in stimulating cytoplasmic CXCR4 expression in vitro. Formation of the biomarker nitrotyrosine was also correlated with CXCR4 expression and lymph node metastasis in vivo. In addition, cytoplasmic CXCR4 expression may serve as a significant prognostic factor for long-term survival in breast cancer.

## Background

Nitric oxide (NO) acts as an intercellular secondary messenger in all mammalian organs, participating in vasodilatation, neurotransmission, and macrophage-mediated immunity [1]. It also possesses metastasis-promoting properties. NO induces vascular endothelial growth factor (VEGF) expression in cancer cells and neovascularization in tumors, which may promote the metastatic ability of tumor cells [2]. Previously we reported that NO induces expression of the lymphangiogenic factor VEGF-C in vitro and in vivo, and may play an important role in lymph node metastasis in breast cancer [3]. The effects of NO are mediated in part by its metabolites, such as peroxynitrite. Peroxynitrite can oxidize and nitrate DNA as well as tyrosine in proteins to produce nitrotyrosine [4]. Thus the presence of nitrotyrosine in tissues has been used as a biomarker for peroxynitrite formation in vivo from NO.

Metastasis of cancer cells is a complex process involving invasion, hemangiogenesis, lymphangiogenesis, trafficking of cancer cells through blood or lymph vessels, extravasations, organ-specific homing, and growth. Recent evidence suggests that metastatic breast cancer cells overexpress CXC chemokine receptor 4 (CXCR4), and that CXCR4 plays a critical role in the homing of cancer cells to specific metastatic sites [5]. The CXCR4 ligand CXCL12 was found to be expressed in liver, bone marrow, lung, and lymph nodes. Furthermore, metastasis of breast cancer cells to regional lymph nodes and lungs in immunodeficient mice were inhibited by a neutralizing antibody against CXCR4 [5]. Previous studies also demonstrated that up-regulated CXCR4 expression in human breast cancer is correlated with lymph node metastasis and unfavorable prognosis [6,7]. However, the mechanisms of regulation of CXCR4 expression are largely unknown. We considered the possibility that NO as an inflammatory stimulant is involved in the expression of CXCR4 because NO has been shown to up-regulate the expression of prometastatic and angiogenic genes including VEGF [2], VEGF-C [3], and VEGF-D [8]. In experimental tumor models, a contributory role of NO in tumor metastasis has also been demonstrated [9]. In addition, signal-activated transcription factor NF-kappa B, which is linked to NO signaling pathways, has been shown to up-regulate the expression of CXCR4 and to mediate CXCL12-induced T cell migration [10,11].

Recently a positive correlation between lymph node metastasis and a recombination of CXCR4, VEGF, and MMP-9 was reported [12]. This correlation may become more important as VEGF had been reported to promote breast carcinoma invasion in an autocrine manner by regulating CXCR4 [13], and at the same time CXCR4 promotes VEGF-mediated tumor angiogenesis [14]. An enhanced-aggressiveness for lymph node metastasis by recombination of VEGF-C and CXCR4 may be also speculated.

In this study, we examined how CXCR4 expression relates to nitrotyrosine formation and lymph node metastasis in human breast cancer tissues, and further investigated whether CXCR4 has any value or relevance for predicting disease outcome. We then showed that incubation of MDA-MB-231 and SK-BR-3 breast cancer cells with an NO donor results in induction of cytoplasmic CXCR4 expression. This induction is significantly inhibited by addition of the NOS inhibitor L-NAME.

## Methods

### Patients and tumor samples

This study was approved by the review board of the Wakayama Medical University Medical Ethics Committee and informed consent was obtained from each of the patients. Archival paraffin-embedded specimens of invasive breast cancer from 113 patients who were diagnosed and treated in the Osaka Police Hospital, Japan, between 1981 and 1992, were selected as described previously [3]. None of these cases had a family history of breast cancer or malignancy in first-degree relatives as determined by questioning at the time of admission for surgery. The patients had received mastectomy with axillary lymph node dissection. All women were apparently free of distant metastasis. All cases received post-operative adjuvant therapy consisting of combination chemotherapy and hormone treatment. The results of immunostaining for ER, PgR, c-erbB-2, p53, and VEGF-C were obtained from our pathological data file [3]. The size of the primary tumor was determined from the surgical specimen. Lymph node metastasis was determined by counting the number of axillary lymph nodes with histological evidence of metastatic breast carcinoma. Histological typing and histological grading were done according to the WHO classification [15], and the Nottingham method (Bloom Richardson) [16]. Patient and tumor characteristics are shown in Table 1. The median age at diagnosis for the 113 patients was 51 years (range, 24–87 years). Fifty-eight percent of the patients were younger than 50 years (n = 65), and 52% (n = 59) of the patients had lymph node metastasis at the time of surgery. Twenty-six percent of the patients had distant organ metastasis during the follow-up period (n = 29).

**Table 1:** The relationship between CXCR4 expression and other parameters

		CXCR4 Positivity		CXCR4 positivity	
Factor		Cytoplasm	p value	Nucleus	p value

Age	<50	28/65 (43%)	0.1298	17/65 (26%)	0.8896
	≧51	28/48 (58%)		12/48 (25%)	
Histology	Ductal	54/104 (52%)	0.1620	28/104 (27%)	0.4432
	Others	2/9 (22%)		1/9 (11%)	
Tumor size	pT1	15/34 (44%)	0.5395	11/34 (32%)	0.3487
	pT2-4	41/79 (52%)		18/79 (23%)	
ER	Negative	24/45 (53%)	0.5671	11/45 (24%)	0.8302
	Positive	32/68 (47%)		18/68 (26%)	
PgR	Negative	27/51 (53%)	0.5729	12/51 (24%)	0.6710
	Positive	29/62 (47%)		17/62 (27%)	
c-erbB-2	Negative	39/81 (48%)	0.6800	22/81 (27%)	0.6387
	Positive	17/32 (53%)		7/32 (22%)	
p53	Negative	36/73 (49%)	0.9445	22/73 (30%)	0.1789
	Positive	20/40 (50%)		7/40 (18%)	
Histological	I and II	31/66 (47%)	0.5695	16/66 (24%)	0.8273
Grade	III	25/47 (53%)		13/47 (28%)	
Nitrotyrosine formation	Low	10/48 (21%)	<0.001	11/48 (23%)	0.6649
	High	46/65 (71%)		18/65 (28%)	
Distant metastasis	Negative	34/84 (40%)	0.0012	22/84 (26%)	1.0000
	Positive	22/29 (76%)		7/29 (24%)	

### Immunohistochemistry

For immunostaining, 4-micrometer thick paraffin sections were de-paraffinized, placed in a solution of 97% methanol and 3% hydrogen peroxide for 5 min, then autoclaved for antigen retrieval. After washing in PBS, the slides were treated for 20 min with Protein Block Serum-free (DAKO Cytomation, Carpinteria, CA, USA). This was followed by an overnight incubation at 4°C in a humidified chamber with a 1:30 diluted anti-human CXCR4 rabbit antibody (Abcam Ltd., Cambridge, UK). After the overnight treatment, to avoid the nonspecific biotin reaction, Histofine Simple Stain MAX PO (NICHIREI, Tokyo, Japan) was used as the second antibody for 60 min according to the manufacturer's instructions. Color was developed using diaminobenzidine with 0.01% hydrogen peroxide. Hematoxylin was used as a counterstain. For the negative control, all reagents except for the primary antibody were used.

The immunohistochemical scoring was performed blindly by 3 pathologists (HY, MT, and YN) who had no clinical knowledge of the patients. The immunostained sections were scanned by light-microscopy, and evaluation of CXCR4 expression was performed according to Cabioglu et al [6]. The intensity, staining percentage, and pattern of staining (nuclear and cytoplasmic) were assessed for CXCR4. The intensity was scored as low, moderate, and strong compared with background staining. The percentages of positive cells were estimated by calculating the ratio of the positively stained invasive tumor cells to the total invasive cells. Nuclear versus cytoplasmic location of expression was also noted in each sample. The staining patterns of tumors for CXCR4 was defined as high cytoplasmic expression (moderate and >50%, or strong and >30% cytoplasmic expression) or predominantly nuclear expression (at least 80% nuclear expression) by using the criteria according to Cabioglu et al [6].

### Cell culture

The MDA-MB-231 and SK-BR-3 breast carcinoma cell lines were purchased from the American Type Culture Collection (ATCC, Rockville, MD, USA). MDA-MB-231 cells were maintained at 37°C in 95% air and 5% CO_2_, as monolayers in tissue culture dishes containing DMEM medium (Invitrogen, Tokyo, Japan) supplemented with 10% heat-inactivated fetal calf serum (FCS) (HyClone, South Logan, UT, USA). SK-BR-3 cells were maintained at 37°C in 95% air and 5% CO_2_, as monolayers in tissue culture dishes containing McCoy's 5A medium (Invitrogen) supplemented with 10% heat-inactivated FCS (HyClone). For the experiments, 6 cm tissue culture plates (Corning Inc, Corning, NY, USA) were seeded with 3 × 10^5 ^cells in 3 ml of medium + 10% FCS. Media were changed on day 3, and when the cells were subconfluent (day 5). 5 mM L-NAME (Sigma-Aldrich, Tokyo, Japan), if administered, was added 2 h before 1 mM DETA NONOate (Cayman Chemical, Ann Arbor, MI, USA). These concentrations of L-NAME or DETA NONOate had no effect on cell viability as measured by the CellTiter 96 Aqueous One Solution Cell Proliferation Assay (Promega, Madison, WI, USA) (data not shown).

### Determination of CXCR4 mRNA expression

After DETA NONOate (half-life of 20 h at 37°C) administration, each cell line was incubated along with DETA NONOate with or without L-NAME for 4, 6, 8, 12, 16, and 24 h. Total RNA was extracted using Trizol (Invitrogen) according to the protocol provided by the manufacturer. After treatment with DNA-free™ DNase (Ambion, Austin, TX, USA), mRNA was reverse-transcribed for single strand cDNA using Oligo-(dT)_20 _primer and Thermoscript (Invitrogen, Tokyo, Japan) as described previously [3]. CXCR4 transcription was measured by quantitative real-time PCR of the resulting cDNA, using universal TaqMan PCR reagents, and an ABI Prism 7000 sequence detector equipped with a 96-well thermal cycler (Perkin-Elmer Applied Biosystems, Foster City, CA, USA). The primer and probe mixtures for CXCR4 and GAPDH were purchased from Perkin-Elmer Applied Biosystems, and PCR was carried out according to the manufacturer's protocol. CXCR4 mRNA expression was quantitated relative to control cells (treated with neither DETA NONOate or L-NAME) based on a real-time PCR standard curve constructed with serially diluted solutions of a CXCR4 cDNA-containing plasmid as templates. All experiments were performed in triplicate, even though amplification results have been shown to be very stable and tube-to-tube-variability very low. Mean values were used for statistical testing. The GAPDH transcript levels of each sample were also monitored; tube-to-tube-variability was very low for these amplifications as well (data not shown).

### Determination of CXCR4 protein expression

For the determination of CXCR4 protein production, each cell line was incubated for 12 h after DETA NONOate administration with or without L-NAME, because the peak time of CXCR4 mRNA expression after DETA NONOate administration was 6–12 h in MDA-MB-231 cells and 8 h in SK-BR-3 cells, and harvested as described above. Cell lysates were prepared using NE-PER™ Nuclear and Cytoplasmic Extraction Reagents (Pierce, Rockford, IL, USA) containing Halt™ Protease Inhibitor Cocktail (Pierce). For Western blot analysis of CXCR4, 40 microgram samples of nuclear extracts or cytoplasmic fractions were separated by electrophoresis on 10–20% SDS polyacrylamide gels and transferred to PVDF membranes by electroblotting as described previously [3]. The membrane was blocked with 5% skim milk in PBS for 1 h at room temperature, incubated overnight with anti-human CXCR4 rabbit antibody (Abcam), rinsed with PBS, and labeled with peroxidase-conjugated anti-rabbit secondary antibody (Dako Cytomation, Denmark) for 1 h at room temperature. The signals were visualized using the LumiGLO Reserve chemiluminescence substrate kit (KPL, Inc, Gaithersburg, MD, USA) and recorded by luminocapture (ATTO, Tokyo, Japan). Anti-beta-2-microglobulin antibody (Dako Cytomation) was used for the internal control of cytoplasmic extract. Anti-transcript factor IID (Upstate, Lake Placid, NY) was used for the internal control of nuclear extract. To compare levels of proteins, the density of each band was measured by densitometry.

### Chemoinvasion assay

Migration assay of each cell type was assayed in a BD Matrigel™ Invasion Chamber 24-well plate (BD Biosciences, Bedford, MA, USA). Briefly, the upper compartment of the chamber was seeded with 5 × 10^3 ^cells, and the lower compartment of the chamber was loaded with medium. Medium in the upper compartment of the chamber was changed (day 3), and 5 mM L-NAME, if administered, was added 2 h before 1 mM DETA NONOate. At the same time, the lower compartment of the chamber was loaded with different concentrations of recombinant human (rh) CXCL12 or without rhCXCL12 (R&D Systems, Minneapolis, MN, USA). The two compartments were separated by Matrigel (10 micrometer thickness and 8 micrometer pore size). Uncoated membranes were used as a control for non-invasive cell migration, in accordance with the manufacturer's directions. After the incubation (day 5), the chamber was removed, and cells that had migrated to the bottom of the membrane were fixed and stained in Cyto Quick Solution (Muto Pure Chemical, Tokyo, Japan) and counted by light microscopy. The percentage of invading cells after incubation (% Invasion) was calculated as (Matrigel)/(Control membrane), according to the manufacturer's protocol.

### Statistics

The effects of drug treatment were analyzed by ANOVA. Fisher's exact test was used to examine the association of CXCR4 expression with age, histological type, tumor size, lymph node metastasis, ER, PgR, c-erbB-2, p53, histological grade, nitrotyrosine levels, and distant organ metastasis, and also to examine the correlation of lymph node metastasis with CXCR4 expression, VEGF-C expression, and a recombination of cytoplasmic CXCR4 and VEGF-C expression. A p value less than 0.05 was considered significant. A software package (JMP IN 5.1.1, SAS Institute, Cary, NC, USA) was used for all statistical testing and management of the database.

## Results

### CXCR4 expression in breast cancer tissue

**Figure 1:**
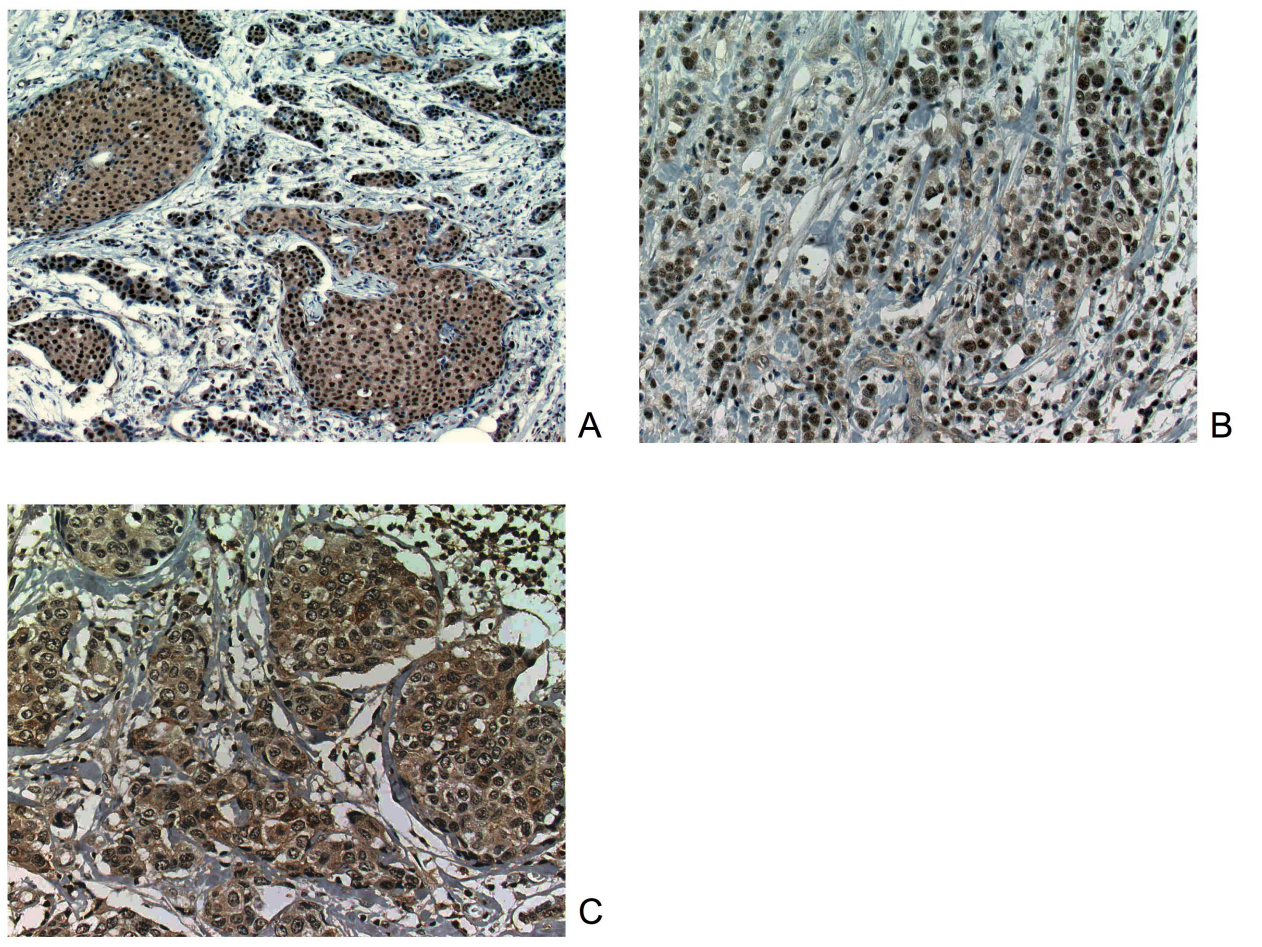
**CXCR4 expression in human breast carcinoma tissue**. (A) The staining pattern of CXCR4 was cytoplasmic staining with a nuclear component. (B) In some cases carcinoma cells showed positive staining predominantly in the nuclei or (C) predominantly in the cytoplasm.

In our series on immunostaining of CXCR4, cytoplasmic staining was more prominent than nuclear staining. Cytoplasmic staining with a nuclear component (Figure 1A) was observed in 20% (23/113) of the tumors, nuclear staining predominantly (Figure 1B) in 5% (6/113), cytoplasmic staining predominantly (Figure 1C) in 29% (33/113), and no staining in 45% (51/113). In addition to tumor cells, CXCR4 staining was also observed in inflammatory cells.

### Cytoplasmic CXCR4 expression is correlated with nitrotyrosine levels and lymph node/distant organ metastasis

We previously reported that nitrotyrosine formation was detected by immunohistochemistry in all invasive breast carcinomas [3]. The intensity of nitrotyrosine immunostaining was evaluated by dividing the cytoplasmic staining reaction into four groups: 1 = weak; 2 = moderate; 3 = strong; and 4 = very strong. The fraction of immunostained cells was scored as follows: 1 = <25%; 2 = 25–50%; 3 = 50–75%; and 4 = >75% of tumor cells showing cytoplasmic staining. These scores were then divided into two groups as low-grade (2–4) and high-grade (5–8) for statistical testing. As we reported previously [3], high-grade nitrotyrosine staining was observed in 57.5% (65/113) of the breast cancer patients, and its expression was significantly correlated with lymph node metastasis (p < 0.001). As shown in Table 1, cytoplasmic CXCR4 expression was correlated with high-grade nitrotyrosine staining (p < 0.001) and distant organ metastasis (p = 0.0012). There was no significant correlation between cytoplasmic as well as nuclear CXCR4 expressions and other clinicopathological factors. As shown in Table 2, lymph node metastasis was correlated with cytoplasmic CXCR4 expression (p = 0.0144), VEGF-C expression (p = 0.0123), and recombination of cytoplasmic CXCR4 and VEGF-C expression (p = 0.0081).

**Table 2:** The relationship between lymph node metastasis and other parameters

	Lymph node metastasis		
Factor	Negative	Positive	p value

Cytoplasmic CXCR4	20/54 (37%)	36/59 (61%)	0.0144
Nuclear CXCR4	16/54 (30%)	13/59 (22%)	0.3941
VEGF-C	39/54 (72%)	54/59 (92%)	0.0123
Cytoplasmic CXCR4 + VEGF-C	18/54 (33%)	35/59 (59%)	0.0081

### Cytoplasmic CXCR4 expression is correlated with patients' survival

Survival analysis was performed on 113 patients and the following variables were examined: cytoplasmic or nuclear CXCR4 expression, tumor size, lymph node metastasis, hormonal status, c-erbB-2, p53, histological grade, and nitrotyrosine formation. As shown in a previous report [3], univariate survival analysis showed that tumor size, lymph node metastasis, ER status, c-erbB-2, and high-grade nitrotyrosine staining were of significant prognostic value for DFS. Lymph node metastasis and high-grade nitrotyrosine staining were of significant prognostic value for OS. In this study, cytoplasmic CXCR4 expression was of significant prognostic value for DFS (p = 0.0020) and OS (p = 0.0002). As shown in Table 3, multivariate Cox regression analysis of all covariates focusing on DFS identified the following as independent significant prognostic factors: tumor size, p = 0.0347: lymph node metastasis, p = 0.0007: and cytoplasmic CXCR4 expression, p = 0.0465. Similarly, lymph node metastasis and cytoplasmic CXCR4 expression were identified as independent prognostic factors for OS (lymph node metastasis; p = 0.0107, cytoplasmic CXCR4 expression; p = 0.0263).

**Table 3:** Results of multivariate Cox regression analysis of disease-free survival (DFS) and overall survival (OS) in 113 breast cancer cases

		Odds ratio (95% CI)		Odds ratio (95%CI)	
Variable		DFS	p value	OS	p value

Tumor size	pT1	1.0 (referent)	0.0347	1.0 (referent)	0.1299
	pT2-4	2.91 (1.08–7.82)		2.08 (0.81–5.39)	
Lymph node metastasis	Negative	1.0 (referent)	0.0007	1.0 (referent)	0.0107
	Positive	5.43(2.03–14.49)		3.32 (1.32–8.32)	
ER	Positive	1.0 (referent)	0.8809	1.0 (referent)	0.3942
	Negative	1.07 (0.46–2.47)		1.50 (0.59–3.83)	
PgR	Positive	1.0 (referent)	0.4759	1.0 (referent)	0.0643
	Negative	1.34 (0.60–3.02)		2.27 (0.95–5.39)	
c-erbB-2	Negative	1.0 (referent)	0.6208	1.0 (referent)	0.7099
	Positive	1.19 (0.59–2.39)		1.16 (0.53–2.56)	
p53	Negative	1.0 (referent)	0.5545	1.0 (referent)	0.5544
	Positive	1.25 (0.60–2.62)		1.30 (0.55–3.07)	
Histological grade	I and II	1.0 (referent)	0.2145	1.0 (referent)	0.1295
	III	1.55 (0.78–3.12)		1.81 (0.84–3.91)	
Nitrotyrosine formation	Low	1.0 (referent)	0.1247	1.0 (referent)	0.0669
	High	2.03 (0.82–5.02)		2.76 (0.93–8.20)	
CXCR4 (cytoplasm)	Negative	1.0 (referent)	0.0465	1.0 (referent)	0.0263
	Positive	2.08 (1.01–4.26)		2.59 (1.12–6.02)	
CXCR4 (nucleus)	Negative	1.0 (referent)	0.7341	1.0 (referent)	0.9446
	Positive	1.15 (0.52–2.55)		1.03 (0.45–2.39)	

### Effects of NO on CXCR4 expression

**Figure 2:**
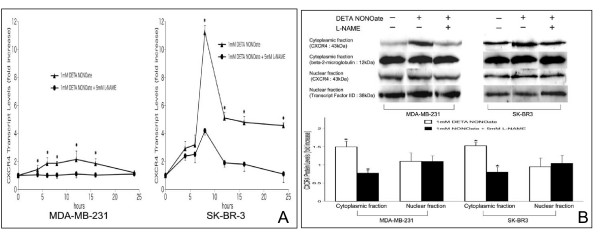
**Effects of NO on CXCR4 expression**. Both MDA-MB-231 and SK-BR3 cells were treated with 1 mM DETA NONOate in the presence or absence of 5 mM L-NAME for various time periods and prepared for (A) real-time RT PCR analysis, and (B) western blot analysis, as described in Materials and Methods. Determinations were performed in triplicate and expressed as the mean of three experiments ± SD. Data are expressed as fold increase relative to control (untreated) cells. * indicates significant difference (p < 0.05) from control and/or L-NAME-treated cells.

To examine the effect of NO on CXCR4 induction, both MDA-MB-231 and SK-BR-3 cells were treated with the NO donor DETA NONOate. As we described previously [3], a significant increase in nitrate/nitrite production was observed in the supernatants after stimulation with DETA NONOate. Pre-treatment of the cells with the NOS inhibitor L-NAME significantly inhibited this increase. As shown in Figures 2A and 2B, significant increases in CXCR4 mRNA and cytoplasmic protein expressions were observed after stimulation with DETA NONOate. Nuclear CXCR4 protein expression was unchanged in both cell lines. Pretreatment with L-NAME substantially inhibited all of these effects of DETA NONOate on CXCR4 expression.

### NO regulates CXCL12-mediated Invasion of breast cancer cells

**Figure 3:**
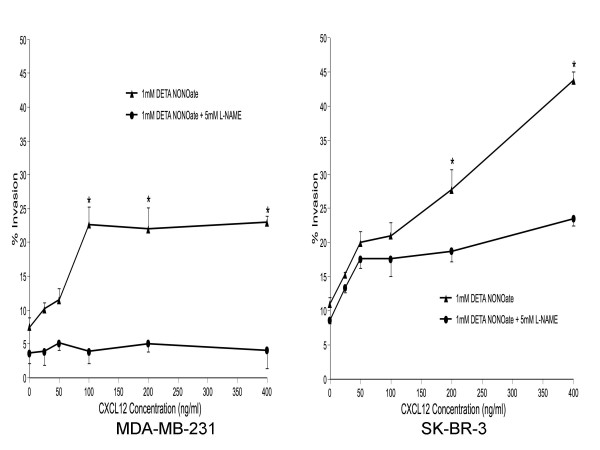
**CXCL12 induced the chemoinvasion of breast cancer cells**. Cells were seeded into the upper compartments of Matrigel Invasion Chambers as described in Materials and Methods. Uncoated or Matrigel-coated membranes separated the upper from the lower compartment containing the indicated concentrations of CXCL12. Cells were treated with DETA NONOate with or without L-NAME, and two days later, cells that had migrated to the bottom of the membrane were stained and counted. The percentage of invasive cells (% Invasion) was calculated as the number of cells penetrating the Matrigel-coated membranes divided by the number penetrating the uncoated membranes. Determinations were performed in triplicate and expressed as the mean of three experiments ± SD. * indicates significant difference (p < 0.05) from L-NAME-treated cells.

To determine the role of NO in cellular response to the CXCR4 ligand CXCL12, we examined the effect of L-NAME on rhCXCL12- and DETA NONOate-induced in-vitro Matrigel invasion by MDA-MB-231 and SK-BR-3 cells (Figure 3). rhCXCL12 produced a dose-dependent increase in invasiveness, which was inhibited in cells pretreated with L-NAME.

## Discussion

NO is involved in various regulatory functions in vivo. It has diverse physiological and pathophysiological roles, such as vasodilatation, neurotransmission, and host defense [1]. As a free radical, NO is reactive and serves as antibacterial and antitumor mediators [17,18]. Also, NO reportedly plays a role in experimental models of tumor cell metastasis [2,9]. Previous reports have shown that NO induces lymphangiogenic factor VEGF-C or VEGF-D expressions in vitro and in vivo, and may play an important role in lymph node metastasis in cancers [3,8,19].

CXCR4 is the physiological receptor for CXCL12, which belongs to a chemokine family that has potent chemotactic activity for lymphocytes. It is well known that peripheral lymphocytes preferentially localize to peripheral lymphoid tissues, such as lymph nodes, which is called the homing phenomenon [20]. Recent evidence suggests that metastatic breast cancer cells overexpress CXCR4 and that this receptor plays a critical role in homing of cancer cells at specific metastatic sites [5].

In this study, immunohistochemistry revealed that cytoplasmic CXCR4 expression was significantly correlated with nitrotyrosine levels, lymph node metastasis, and distant organ metastasis in human breast cancer. The NO donor DETA NONOate induced cytoplasmic expression of functional CXCR4 protein expression in breast cancer cell lines. These results may support a possible connection between cytoplasmic CXCR4 expression and nitrotyrosine formation in human breast cancer. Consistent with up-regulation of CXCR4 expression by NO, 71% of the high-nitrotyrosine patients and only 21% of the low-nitrotyrosine patients were positive for cytoplasmic CXCR4. Although this is lower than the overall percentage of cytoplasmic expression of CXCR4 (49%), it indicates that other factors are also involved in regulating the expression of this receptor. As it has been previously reported that NF-kappa B [10], c-erbB-2 [21], or hypoxia-inducible factor 1 [22] induce CXCR4 expression, these factors may account for CXCR4 positive cases with low levels of nitrotyrosine. In our study, contrary to other reports, there was no significant correlation between CXCR4 expression and c-erbB-2 positivity [7,21]. It has been reported that using anti-CXCR4 monoclonal antibodies may result in an incomplete detection of CXCR4 molecules because of the multiple CXCR4 conformations [23], which may account for the discrepancy between our results using a polyclonal antibody and earlier studies using monoclonal antibodies. Furthermore, lymph node metastasis was correlated with cytoplasmic CXCR4 expression, VEGF-C expression, and recombination of cytoplasmic CXCR4 and VEGF-C expression in this study. Recombination of cytoplasmic CXCR4 and VEGF-C expression was correlated with lymph node metastasis more strongly than cytoplasmic CXCR4 only or VEGF-C only. Similar to the relationship between VEGF and CXCR4, CXCR4 may be involved in promoting VEGF-C-mediated tumor lymphangiogenesis or invasiveness of cancer cells.

In addition, the NO donor DETA NONOate induced CXCR4 mRNA and cytoplasmic protein expression in the MDA-MB-231 and SK-BR-3 breast cancer cell lines. In a chemoinvasion assay, CXCL12-induced invasiveness was observed with both cell lines after treatment with DETA NONOate. All of these responses were significantly inhibited in the presence of the NOS inhibitor L-NAME. A significant increase in nitrate/nitrite production in the supernatants after stimulation with DETA NONOate was also observed, and treatment of cells with L-NAME substantially inhibited this increase as well. Our results suggest that cytoplasmic CXCR4 expression may be regulated by NO in breast cancer cells. In this study, although CXCR4 mRNA expression in SK-BR-3 cells responded well to NO, CXCR4 protein expression was not comparably altered. Inasmuch as SK-BR-3 cells may be more sensitive in its response to NO than MDA-MB-231 cells, an explanation of the differential response in SK-BR-3 cells eludes us, and remains a subject for future studies. We performed the migration assay without the use of Matrigel, however, there was no up-regulation of NO-induced migration activity (data not shown). This may be due to a previous report which found that NO decreased RhoA activity [24], which is a well-known activator of cancer cell motility. Because CXCR4 enhances cancer invasiveness by matrix metalloprotease-13 [25], which is important for invasion of cancer cells by degrading extracellular matrix, this data may support our results of enhanced-invasiveness via NO-CXCR4 signaling.

Another question of practical importance was whether measurement of cytoplasmic CXCR4 expression has any value or relevance with respect to predicting the disease course in breast carcinomas. In our results, survival curves determined by the Kaplan-Meier method and univariate analysis demonstrated that cytoplasmic CXCR4 expression was negatively associated with both DFS and OS. Furthermore, multivariate analysis using the Cox stepwise regression analysis demonstrated that cytoplasmic CXCR4 expression was still correlated with poor DFS and OS after consideration of other prognostic factors. Therefore, cytoplasmic CXCR4 expression appears to be a reliable prognostic biomarker. Although we reported previously that high-grade nitrotyrosine formation may become a useful prognostic indicator for OS [3], high-grade nitrotyrosine staining was not identified as an independent prognostic factor for OS in this study (p = 0.0669). It may be influenced by cytoplasmic CXCR4 positivity.

Cytoplasmic and nuclear CXCR4 expression in cancer cells has been described in various cancers [6,26,27]. In our study, cytoplasmic CXCR4 expression was thought to be critical for lymph node metastasis and the patients' poor prognosis in comparison with nuclear CXCR4 expression. CXCR4 localization at the plasma membrane and intracellular vesicle (cytoplasm) were observed in leukocyte cell lines with enforced CXCR4 expression and CXCL12 induced polarization of CXCR4 to the edge of migrating leukocyte cells [28]. Thus, cytoplasmic CXCR4 expression in cancer cells may be more important for migration of cancer cells, leading in turn to lymph node metastasis and poor prognosis. Although nuclear CXCR4 expression occurs in normal and cancer tissues [7], its function is unknown. As it has been reported that a splice variant of one kind of chemokine lacking the signal peptide is translocated in the nucleus [29], nuclear CXCR4 accumulation may lack a signal peptide and may not be functional.

Recently Balabanian et al demonstrated that CXCL12 can also be a ligand for CXCR7 [30]. Previous report also showed that CXCR7 expression in tumor cells supports cell growth, survival advantage, and increased adhesion properties, and also causes in vivo tumor growth in animal models [31]. Since CXCL12 is expressed preferentially in lymph nodes, this may support our data that CXCR4 expression was significantly correlated with lymph node metastasis in human breast tumor samples. As CXCR7 expression also promotes cancer metastasis in breast cancer [32], it would be important to investigate the correlation between CXCR4 and CXCR7 in breast cancer in future studies.

## Conclusion

Nitric oxide induces cytoplasmic expression of functional CXCR4 expression in vitro and in vivo. Cytoplasmic CXCR4 expression is significantly associated with lymph node metastasis and high nitrotyrosine levels. NO regulation of CXCR4 expression may play an important role in lymph node metastasis in breast cancer. Furthermore, cytoplasmic CXCR4 expression may serve as a significant prognostic factor for long-term survival in breast cancer.

## Abbreviations

CXCR4: CXC chemokine receptor 4; NO: Nitric Oxide; VEGF: Vascular Endothelial Growth Factor; DMEM: Dulbecco's Modified Eagle Medium; FCS: Fetal Calf Serum; L-NAME: N^G^-nitro-L-arginine methyl ester; DETA NONOate: (Z)-1-[2-(2-Aminoethyl)-N-(2-ammonioethyl)amino]diazen-1-ium-1,2-diolate; NOS: NO synthase; PBS: phosphate-buffered saline; RT: reverse transcription; PCR: polymerase chain reaction; GAPDH: glyceraldehyde-3-phosphate dehydrogenase; ER: estrogen receptor; PgR: progesterone receptor; OS: overall survival; DFS: disease-free survival.

## Competing interests

The authors declare that they have no competing interests.

## Authors' contributions

HY conceived the study, participated in the design of the study, conducted and evaluated both the immunostainings and the in vitro assay, performed the statistical analysis and drafted the manuscript. MT contributed to the design of the study, evaluated the immunostainings, and helped to draft the manuscript. KY and MN participated in the design and coordination of the study. RK and TS contributed to the design of the study and interpretation of the results. YN participated in the design, evaluated both the immunostainings and the in vitro assay, and helped to draft the manuscript. All authors read and approved the final manuscript.

## Pre-publication history

The pre-publication history for this paper can be accessed here:


